# Acetone as Indicator of Lipid Oxidation in Stored Margarine

**DOI:** 10.3390/antiox10010059

**Published:** 2021-01-06

**Authors:** Sarah Fruehwirth, Sandra Egger, Thomas Flecker, Miriam Ressler, Nesrin Firat, Marc Pignitter

**Affiliations:** 1 Department of Physiological Chemistry, Faculty of Chemistry, University of Vienna, 1090 Vienna, Austria; sarah.f.fruehwirth@univie.ac.at (S.F.); Sandra.Egger@senna.at (S.E.); 2 Senna Nahrungsmittel GmbH & Co KG, 1140 Vienna, Austria; Nesrin.Firat@senna.at; 3 Josef Ressel Center for Development of Comprehensive Analytical Tools for the Pharmaceutical Industry, Department of Biomedical Science, FH JOANNEUM, 8020 Graz, Austria; flecker.th@gmail.com (T.F.); Miriam.Ressler@fh-joanneum.at (M.R.)

**Keywords:** acetone, HS-SPME-GC-MS, margarine, lipid oxidation

## Abstract

Margarine contains a minimum of 80% fat and is therefore prone to lipid oxidation. While lipid oxidation in vegetable oils and *o*/*w* emulsions has been thoroughly investigated, studies about the oxidative stability and the identification of potential indicators of lipid oxidation in margarine are scarce. To evaluate the oxidative stability and to indicate the progress of lipid oxidation, four different types of industrial margarine (M1–M4), which differed in their composition of the minor ingredients and the oil phase, were stored at 15 °C for 180 days and analyzed at days 0, 1, 7, 14, 28, 56, 99, and 180 regarding peroxides, conjugated dienes, oxidized triacylglycerols, and volatiles. The peroxide value and the conjugated dienes increased up to 4.76 ± 0.92 meq O_2_/kg oil and 14.7 ± 0.49 in M2, respectively. The oxidative stability decreased by a maximum of 50.9% in M4. We detected three different epoxidized triglycerides—TAG54:1 (O), TAG54:2 (O) and TAG54:3 (O)—in M3. Acetone could be identified, for the first time, as lipid oxidation product in stored margarine by headspace-solid-phase microextraction-gas chromatography-mass spectrometry (HS-SPME-GC-MS). It increased in all types of margarine during storage by a maximum of 1070 ppb in M2. Acetone might be used as a new indicator for lipid oxidation in margarine.

## 1. Introduction

Margarine is a low-cost alternative to butter and other fat-soluble spreads. It helps to add volume and texture to bakery products and is thus considered as suitable ingredient for bakery products, like pastries, doughnuts, and cookies, by the industry. Further, margarine is recognized as a healthy alternative for cooking and food preparation by health professionals and consumers and makes an important nutritional contribution to the diet by representing a source of essential fat-soluble vitamins, by being low in saturated fatty acids and due to the absence of cholesterol [1]. By definition, margarine has to contain a minimum of 80% fat by weight, but any edible oil or fat source may be used for its manufacture [2]. Therefore, margarine is regarded as an emulsion of water droplets in oil (*W*/*O*) [3]. Due to the high content of fats, margarine is prone to lipid oxidation. Lipid oxidation in emulsions is usually influenced by many factors, such as the initial concentration of oxygen in the water phase and in the fat blend, the presence of prooxidants and antioxidants, the structure of the interface, the presence of oxygen in the package, and the storage conditions [4]. Hence, lipid oxidation is a major cause of deterioration of margarine during its manufacture, which is mostly noticeable by the development of unpleasant odors [5]. These odors do not only mark oxidative degradation products with potentially toxic characteristics [6] but lead also to the rejection of the products by consumers [5].

As studies concerning lipid oxidation in margarine are scarce, there is a growing interest in evaluating the oxidation process during storage of margarine to determine the accurate shelf-life of the products [6], thereby promoting not only healthier products but also decreasing food waste [7].

Nogala-Kalucka and Gogolewski [8] stored margarine at 4 °C and 20 °C for 136 days. They analyzed the composition of fatty acids, the peroxide value and the tocopherol content during storage and showed that the margarine stored at 4 °C maintained its high nutritive value, whereas the tocopherols in margarine stored at 20 °C decomposed considerably. Zaeroomali et al. [7] stored table margarine for 90 days at 15 °C and investigated its physicochemical properties. They showed that the peroxide value increased, and the oxidative stability decreased but acidity, refraction index, iodine value, and moisture content remained constant. The same was shown by Maskan et al. [6] who stored margarine at 4 °C and 14 °C and analyzed increasing peroxide values and decreasing oxidative stability by the Rancimat method.

Although, several storage trials have been conducted that all determine raising peroxide values with increasing storage time, an official limit for the disposal of oxidized margarine is still missing. Hence, it would be beneficial not only to analyze the oxidation status of margarine with different established methods but also to screen for new possible indicators that can rapidly detect the progress of lipid oxidation in margarine.

Consequently, ketones, which have been shown to form during lipid oxidation from an unsaturated site in triacylglycerols (TAG) [9], might have the capability to rapidly and simply indicate the oxidation status of margarine. Breath acetone, the simplest ketone, has already been described as a biomarker for lipid oxidation [10] in humans.

Therefore, the aim of this work was to evaluate if acetone might be a novel indicator for secondary lipid oxidation in margarines. Hence, four different and commercially available margarines were stored at 15 °C for 180 days and a headspace solid-phase microextraction-gas chromatography-mass spectrometry (HS-SPME-GC-MS) method for the measurement of acetone was developed. Further, the results of the HS-SPME-GC-MS method were compared with well-known and established methods like the peroxide value, conjugated dienes, the Rancimat method and the measurement of oxidized triacylglycerols (oxTAG) to confirm its applicability.

## 2. Materials and Methods

### 2.1. Chemicals and Materials

All chemicals were purchased from Merck (Vienna, Austria), Carl Roth (Karlsruhe, Germany) or VWR International GmbH (Vienna, Austria). Solvents used for chromatography were HPLC grade.

### 2.2. Margarine Samples and Study Design

There were four different, commercially available, margarines (M1–M4) provided by Senna GmbH & Co KG (Vienna, Austria) directly after production. Margarines were produced in 2.5 kg bars and wrapped in aluminum foil. Four bars were packed in one carton and stored at 15 °C for 180 days. Samples were taken at days 0, 1, 7, 14, 28, 56, 99, and 180. For each of the eight days, a separate carton of M1, M2, M3, and M4, containing four equal margarine bars, were stored. At each day 50 g margarine were taken from the middle of four different bars (n = 4) and stored under argon atmosphere at −80 °C till sample preparation.

The composition of the different margarines is given in Table 1. Refined oils were used to produce the different margarines.

### 2.3. GC/FID Analysis of the Fatty Acid Composition

Fatty acids were analyzed as their respective fatty acid methyl esters (FAME) via GC and FID (Agilent 7890A, Vienna, Austria) in all margarines. Sample preparation and GC and FID analysis were done according to the method C-VI 10a from the German Society for Fat Science (DGF) [11]. In brief, 50 g margarine were heated at 80 °C in an oven (DRY-Line^®^, VWR International GmbH) until phase separation. A total of 200 µL of the fat phase was combined with 2 mL of isooctane in a tube. Then, 100 µL of a methanolic potassium hydroxide solution (1.2 M) were added and the tube was shaken for 30 s. A drop of a 0.1% methyl orange solution was added as indicator and the pH was adjusted with 1 N HCl below four. The upper phase was used for analysis. Samples were analyzed in quadruplets.

GC and FID analysis was performed using a HP-88 (88% cyanopropyl) aryl polysiloxane column (100 m × 250 µm × 0.2 µm, Agilent Technologies Österreich GmbH, Vienna, Austria.). The temperature gradient was programmed as follows: 100 °C for 4 min, then increase to 170 °C within 6 min and to 240 °C within 10 min. The sample was injected (2 µL) with a split-ratio of 5:1 and a split-flow of 10 mL/min at 250 °C. Carrier gas consisted of hydrogen (2 mL/min). Gases for the FID were nitrogen (25 mL/min), hydrogen (30 mL/min), and synthetic air (300 mL/min).

For quantitation, the percental distribution of the fatty acids was evaluated by comparing the area of each FAME with the total area of all FAMEs. Retention times were determined using standards.

### 2.4. Peroxide Value

The peroxide value was measured according to the method of Wheeler [12] and DGF C-VI 6a [11] by using an automatic titration unit (Titrino plus 848, Metrohm Inula GmbH, Vienna, Austria) with electrochemical endpoint determination. In brief, 2.5 g margarine were melted at 50 °C and dissolved in 20 mL acetic acid and isooctane (3:2, *v*/*v*). A total of 200 µL of a saturated potassium iodide solution was added. The sample was stirred and 80 mL bidestilled water were added.

The titrant solution (1 mM Na_2_S_2_O_3_) was added automatically until the equivalence point was reached. The peroxide value was then calculated automatically from the titration unit and indicated in meq O_2_/kg oil. Samples were measured in quadruplets.

### 2.5. Conjugated Dienes

Conjugated dienes were determined according to Pegg [13] and the DGF method C-VI 6 [11] with slight modifications. In brief, 3 g of margarine were melted on a heating plate at 50 °C. Then, 0.01 g were dissolved in 10 mL of isooctane and measured at 233 nm in a photometer (ONDA UV-30 SCAN Spectrophotometer, Giorgio Bormac s.r.l., Carpi, Italy) against isooctane as a blank.

The amount of conjugated dienes was calculated according to Pegg [13]. Samples were measured in quadruplets.

### 2.6. Analysis of Oxidized Triacylglycerols by Targeted LC-MS/MS

First, amber glass vials were cleaned to remove all traces of lipids and lipid oxidation-promoting agents [14]. Then, polar oxidation products were separated from triacylglycerols in margarine samples using Sep-Pak silica columns (Strata SI-1 Silica, 500 mg, Phenomenex, Aschaffenburg, Germany) according to the protocol described by Márquez–Ruiz et al. [15] with some modifications according to Fruehwirth et al. [16]. In brief, the Sep-Pak silica columns were conditioned before use by rinsing with 10 mL of petroleum ether and diethyl ether (90/10) and 50 mg of the margarine, dissolved in 1 mL of petroleum ether and diethyl ether (90/10), were loaded on the column. The nonpolar fraction was eluted with 2 mL petroleum ether and diethyl ether (90/10) and discarded. The second fraction which contained the polar compounds was eluted with 2 mL diethyl ether. The polar fraction was evaporated with nitrogen and stored under argon atmosphere at −40 °C until analysis.

Prior to analysis, the samples were dissolved in 0.5 mL 2-propanol and injected (4 µL) into a LC-MS system (LCMS-8040, Shimadzu, Korneuburg, Austria) and separated on a C18 column (trimethylsilane endcapped Luna C18, 150 × 2.1 mm, 3 µm, Phenomenex, Aschaffenburg, Germany). This column can be used when maximum retention of non-polar analytes is desired and is recommended for the separation of hydrophobic compounds. The mobile phase consisted of acetonitrile and H2O (60/40) with 0.1% formic acid and 10 mM ammonium formate (A) and acetonitrile and isopropanol (20/80) with 0.1% formic acid and 10 mM ammonium formate (B).

The following HPLC gradient was used: 0–8 min with 60% B to 100% B, 8–28 min 100% B, 28–30 min 100% to 60% B and 30–35 min 60% B at a flow rate of 0.5 mL/min. The HPLC and MS settings were applied according to Grüneis et al. [14].

Calibration curves for quantitation were performed using a glyceryltriheptadecanoate standard (R^2^ = 0.9944) [14]. The limit of detection (LOD) and the limit of quantitation (LOQ) were determined using the blank determination method [17]. The LOD was 4.80 nM and the LOQ was 15.7 nM for hydroperoxidized as well as epoxidized TAGs.

### 2.7. Oxidative Stability Determined by Rancimat Method

The oxidative stability of margarine was determined using the Rancimat method [18,19]. Therefore, the oxidation induction time (OIT, in hours) was evaluated with the Rancimat apparatus (Metrohm 743, Herisau, Switzerland). For all analyses, 20 g margarine were melted in an oven (DRY-Line^®^, VWR International GmbH) at 80 °C for 25 min. M2, which contained milk, had to be clarified with 3 g sodium sulfate decahydrate during the heating. Then, Rancimat vessels containing 3 g margarine were used for the analysis and an air rate of 10 L/h was applied. Each measuring vessel contained 60 mL distilled water. The OIT was evaluated using a temperature of 120 °C. Samples were measured in quadruplets.

### 2.8. Headspace SPME-GC-MS Analysis

Volatiles of margarine were evaluated by HS-SPME-GC-MS according to Dadali et al. [20] and Shiota et al. [21]. Margarine samples (1 g) were equilibrated at room temperature for 30 min and filled into the SPME vials using a 2 mL syringe to prevent sample materials from sticking to the walls of the vials. Volatiles of margarine were extracted for 40 min at 50 °C using a 50/30 µm divinylbenzene/carboxen/polydimethylsiloxane SPME fiber (2 cm) on a PAL3 RSI (CTC Analytics, Zwingen, Switzerland) with sample pre-conditioning step of 10 min at 50 °C.

GC-MS analysis was performed using an Agilent 7890 gas chromatograph (Vienna, Austria) coupled with an Agilent 5977 single quadrupole mass spectrometer (Santa Clara, CA, USA). An Agilent VF-624 MS capillary column (60 m × 250 µm i.d., 1.4 µm film thickness) was used for the separation of the analytes. The SPME-fiber was desorbed for 1 min in the GC inlet port which was operated in splitless mode at a temperature of 260 °C. The initial temperature of the column oven program was 40 °C which was held for 5 min. The temperature was increased to 240 °C at a heating rate of 8 °C/min. A second ramp with a rate of 15 °C/min brought the temperature to 270 °C which was held for 8 min. The carrier gas was helium (99.999%) at a constant flow of 1.2 mL/min which resulted in an average flow velocity of 18.768 cm/s. The transfer line to the MS was kept at a temperature of 320 °C. The electron energy in the electron impact source was 70 eV and the filament current 150 µA. The ion source was kept at 230 °C and the quadrupole at 150 °C. The *m*/*z* acquisition range of the MS was 30 to 550.

The peaks obtained were identified based on Wiley–National Institute of Standards and Technology library search with subtraction of corresponding sample blanks and an area threshold of at least 5000 counts. All identified compounds are summarized within the heatmap as average of two independent replicates.

In order to determine storage-dependent alterations of acetone and hexanal, non-stored margarine samples were compared to stored samples using GC-MS data of three independent replicates. Quantification of the target compounds was carried out with the respective *m*/*z* ions at 43 and 58 for acetone and *m*/*z* 56 and 72 for hexanal, by applying standard addition experiments. Spiking concentrations were individually selected for each sample type considering the increasing amount over the period of time. Accordingly, the neat samples were enriched with a maximum of twice the estimated compound concentration, by adding the respective amount dissolved in 25 mg glyceryl trioctanoate to 1 g margarine. Glyceryl trioctanoate without the target compounds was added to neat samples corresponding to spiking level 0. For each of the neat as well as the spiked samples, three independent replicates were prepared. In order to estimate the precision of the analysis a 95% confidence interval was used, considering the experimentally determined concentration of the analyte within the respective sample. To monitor sample specific effects during HS-SPME, 1-pentanol was added at a concentration of 500 μg/L.

### 2.9. Statistical Analysis

Data were analyzed using Microsoft Excel and SigmaPlot 14.0 (Synstat Software GmbH, Erkrath, Germany) and are shown as mean ± SD of three to four independent experiments (n = 3–4). Outliers were excluded via Nalimov test. Statistical analysis was performed via one-way analysis of variance (ANOVA) and Holm–Sidak post-hoc test. If data did not exhibit normal distribution, results were determined via Kruskal Wallis ANOVA followed by Student–Newman–Keuls’ post-hoc test. The Spearman’s rank-order correlation analysis was performed between the acetone concentration in the stored margarine samples and the corresponding content of unsaturated fatty acids, conjugated dienes, peroxide value and the OIT since data did not follow a normal distribution. Multivariate statistical analysis was performed on spectral data using the Agilent Mass Profiler Professional 15.1. For the sample heatmap, mass traces of the target compounds were aligned based on their overall intensity within the whole dataset (n = 2).

## 3. Results and Discussion

### 3.1. Fatty Acid Composition of Margarines

**Figure 1:**
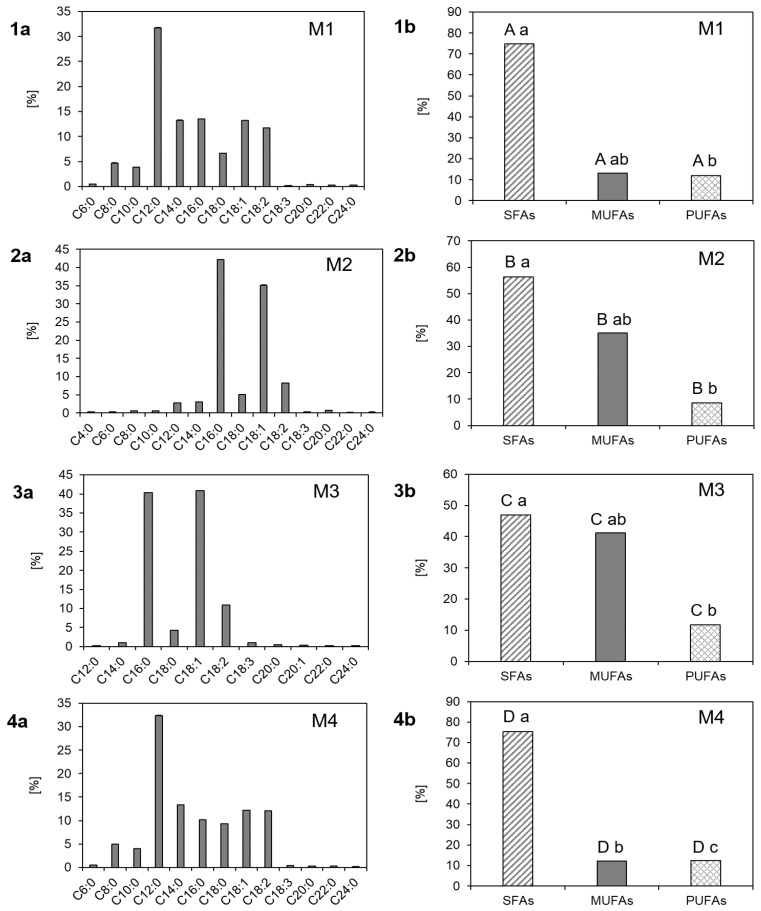
(**a**) Fatty acid composition and (**b**) content of SFAs, MUFAs and PUFAs in margarines (1) M1, (2) M2, (3) M3 and (4) M4. Data are depicted as mean + SD (n = 4). Statistically significant differences between the SFAs, MUFAs, and PUFAs within each margarine are indicated with different lowercase letters. Capital letters indicate the significant differences between the SFAs, MUFAs, and PUFAs of the different margarines M1–M4 (one-way ANOVA, Holm–Sidak, *p* < 0.05).

The four different margarines were characterized regarding their fatty acid composition. It could be shown (Figure 1) that saturated fatty acids (SFA), monounsaturated fatty acids (MUFA) and polyunsaturated fatty acids (PUFAs) differed significantly (*p* < 0.05) between the different margarines. Still, M1 and M4 exhibited a similar fatty acid composition with coconut oil being the main oil content in both margarines with 54% and 56%, respectively (Table 1). Therefore, both margarines showed high amounts of lauric acid (C12:0) and myristic acid (C14:0), which are typical for coconut oil [22]. M1 exhibited 31.7% ± 0.09% lauric acid and 13.2% ± 0.17% myristic acid, whereas M4 exhibited 32.4% ± 0.04% lauric acid and 13.4% ± 0.01% myristic acid. Further, both margarines (M1 and M4) showed a similar content of oleic acid (C18:1) with 13.2% ± 0.05% and 12.2% ± 0.04%, respectively. As both margarines contained sunflower oil, they exhibited 11.7% ± 0.05% and 12.1% ± 0.04% linoleic acid, respectively [23]. The sunflower oil, which was used to produce margarines M1 and M4, was of the low oleic and high linoleic type. Even though it is known that high oleic sunflower oil is more stable than common sunflower oil [24], it is rarely used to produce margarines. The presence of MUFAs has been shown to largely influence the heat resistance of sunflower oil, even more than the presence of antioxidants, such as α-tocopherol [24]. Giuffrè et al. [25] studied the influence of high temperature and heating time on the nutritional properties of low oleic sunflower oil and concluded that the most appropriate heating temperature for this oil is ranging between 180 and 210 °C and that it can be heated for 120 min. These findings may be important for the use of margarine for cooking and baking.

M2 contained 61% palm oil and therefore exhibited high contents of palmitic acid (C16:0) and oleic acid with 42.1% ± 0.09% and 35.1% ± 0.04%, respectively [26]. Palm oil contains a unique lipid profile and is the only vegetable oil that contains almost 50% of saturated C16:0 fatty acids and 50% of C18:1 unsaturated fatty acids [27]. Therefore, it exhibits a high oxidative stability and contributes to an improved shelf life for processed foods [27]. Due to its high smoke point it is even usable for frying [28]. Further, 8% anhydrous milk fat were added to M2, which contributed to the high amounts of palmitic acid and oleic acid. Butter consists of 99.3% cow’s fat which consists of 30% palmitic acid and 22.8% oleic acid [29].

Like M2, M3 contained high amounts of palm oil with 57% and 12% palm stearin. Thus, M3 exhibited high amounts of palmitic acid and oleic acid [26] with 40.4% ± 0.04% and 40.9% ± 0.07%, respectively.

Regarding SFAs, margarine M4 contained the highest amount with 75.4% ± 0.01% followed by M1 with 74.9% ± 0.02% and M2 and M3 with 56.3% ± 0.06% and 46.9% ± 0.05%, respectively. M3 contained the highest amounts of MUFAs with 41.2% ± 0.00%, followed by M2 with 35.1% ± 0.01% and M1 and M4 with 13.2% ± 0.00% and 12.2% ± 0.00%, respectively. Regarding PUFAs margarines M1, M3 and M4 exhibited similar contents with 11.9% ± 0.02%, 11.8% ± 0.00% and 12.5% ± 0.02%, respectively, whereas M2 exhibited lower contents with 8.6% ± 0.02%.

### 3.2. Oxidative Stability of Margarines during Storage

The oxidative stability of margarine during a storage period of 180 days at 15 °C was analyzed using well-known and established methods like the peroxide value, conjugated dienes and the Rancimat method. Furthermore, oxTAGs were determined by LC-MS.

#### 3.2.1. Peroxide Value

The peroxide value is defined as the amount of oxygen taken up by the fat or oil upon formation of peroxides and should be below 15 meq O_2_/kg oil for cold pressed and virgin oils and below 10 meq O_2_/kg oil for refined oils according to the Codex Alimentarius [30]. Though, the Codex Alimentarius defines the limits for cold pressed and refined oils, it does not define any limit for the formation of peroxides in margarine.

Comparing the different margarines during storage, it could be analyzed that the peroxide value increased in all samples (Table 2) as expected from literature [7,8]. M1 exhibited the lowest peroxide value directly after production with 0.33 meq O_2_/kg oil, followed by M4 and M3 with a peroxide value of 0.40 and 0.86 meq O_2_/kg oil, respectively. M2 had not only the highest value after production but increased as well to the highest value after a storage of 180 days as the peroxide value raised from 1.10 meq O_2_/kg oil to 4.76 meq O_2_/kg oil. This could be explained by M2 consisting of 8% anhydrous milk fat which had to be heated before the production. Interestingly, M2 had the lowest increase in peroxides during storage as it changed by about 333% between day 0 and day 180. M3 changed by about 362% whereas M1 and M4 changed by about 567% and 583%, respectively. This could be explained by the different oils used to produce the different margarines (Table 1, Figure 1). M1 and M4 contained the highest amounts of SFAs, which explains their low peroxide values at day 0. The least pronounced increase in the peroxide value in M2 during the 180 days of storage might be explained by the lowest amount of PUFAs in M2. To verify the results of the peroxide value, a second method for the determination of primary lipid oxidation products was applied.

#### 3.2.2. Conjugated Dienes

Conjugated dienes are widely accepted for the evaluation of lipid oxidation [31]. They are generated by the oxidation of polyunsaturated fatty acids (PUFAs) and are therefore used to assess the oxidative rancidity of vegetable oils [32].

The results of the conjugated dienes in the different margarines are given in Table 2. The conjugated dienes in the samples increased during storage, as shown before with the peroxide value. Further, M1 and M4 exhibited lower contents of conjugated dienes at day 0 than M2 and M3, whereas conjugated dienes in M2 and M3 increased less than in M1 and M4 during storage. Both results confirmed the outcome of the peroxide value.

As both the conjugated dienes and the peroxide value determine primary oxidation products only, the Rancimat method was used to analyze secondary oxidation products.

#### 3.2.3. Oxidative Stability Measured by Rancimat Method

Due to the instability of primary oxidation products, secondary lipid oxidation products are formed which can further decompose and result in the formation of volatile products [31]. The Rancimat method determines the oxidative stability by detecting these volatile products which are formed after heating of the samples.

The OITs of the different margarine samples are given in Table 2. It could be shown that the OIT decreased in all margarines during storage. M2 and M3 showed a higher oxidative stability with 13.0 ± 0.23 h and 11.7 ± 0.37 h, respectively, than M1 and M4 with an OIT of 10.3 ± 0.22 h and 10.7 ± 0.09 h, respectively. Further, the OIT of M2 and M3 decreased only by about 38.8% and 34.1%, respectively, whereas the OIT of M1 and M4 decreased by about 46.1% and 50.9%, respectively.

However, commonly applied methods, like the determination of the peroxide value, conjugated dienes and the Rancimat method, focus on the measurement of conjugated dienes, hydroperoxides and their scission products, but epoxides are not recorded [14], although it has been demonstrated that epoxides might even be more harmful than lipid aldehydes [33]. Therefore, hydroperoxidized and epoxidized triacylglycerols were analyzed additionally.

#### 3.2.4. Determination of oxTAGs by LC-MS

By analyzing the margarine samples during storage, only epoxidized TAGs could be detected as hydroperoxidized TAGs were below LOD (Table 3). This was confirmed by the results of a previous work, where the concentration of epoxides in margarine was much higher than the concentration of hydroperoxidized TAGs [14].

Ibargoitia et al. [34] were also able to detect epoxides in margarine when heated to high temperature. In the current study, epoxides were only determined in M2 and M3. These margarine samples consisted mainly of palm oil, being rich in oleic acid, whereas M1 and M4 were prepared using mainly coconut oil, which is low in unsaturated fatty acids, explaining the detection of epoxides in M2 and M3 only. In M2 two epoxides—TAG54:1 (O) and TAG54:2 (O)—were formed. Both epoxides decreased during storage. The epoxide TAG54:1 (O) decreased from 0.58 ± 0.03 mg epoxide/kg margarine to 0.24 ± 0.06 mg epoxide/kg margarine, whereas the epoxide TAG54:2 (O) decreased from 0.19 ± 0.01 mg epoxide/kg margarine to 0.08 ± 0.06 mg epoxide/kg margarine. In M3 three epoxides—TAG54:1 (O), TAG54:2 (O) and TAG54:3 (O)—were formed, which increased during storage. The epoxide TAG54:1 (O) increased from 1.06 ± 0.10 mg epoxide/kg margarine to 1.59 ± 0.33 mg epoxide/kg margarine, the epoxide TAG54:2 (O) increased from 0.77 ± 0.11 mg epoxide/kg margarine to 1.32 ± 0.33 mg epoxide/kg margarine and the epoxide TAG54:3 (O) increased from 0.07 ± 0.04 mg epoxide/kg margarine to 0.27 ± 0.16 mg epoxide/kg margarine. The difference in the behavior of epoxides, which decreased in M2 but increased in M3, might be explained by the presence of anhydrous milk fat, which was only present in M2, and the different reaction pathways that can lead to the formation of epoxides. Epoxides can be generated either independently of hydroperoxide formation as primary oxidation products [14,35] or by rearrangement of alkoxyl radicals as secondary lipid oxidation products [9]. Therefore, it might be possible that the epoxides in M2 were formed by hydrogen abstraction-independent pathways of early lipid oxidation [14] and that epoxides in M3 were formed as secondary lipid oxidation products from decomposition of hydroperoxides [9]. Thereby, alkoxyl radicals could have been formed through decomposition of hydroperoxides, that further produced epoxides through rearrangement [9], leading to an increase in epoxides in M3. In a recent study, epoxy fatty acids were reported to be formed in palm olein after heat treatment for 24 h [36]. It could be shown that epoxystearates were generated from oleate and epoxyoleates from linoleate. It is known that epoxides are formed by replacing a double bond with an epoxide [9]. Due to the high abundance of MUFAs in M2 and M3, the detection of epoxidized TAGs with one and two double bonds was expected. In M3, which contained a higher amount of PUFAs than M2, an epoxidized TAG with 3 doubled bonds could be quantified.

Ibargoitia et al. [34] stated that monoepoxides derived from linoleic fatty acids deserve special attention, as they are supposed to be toxic. This is of importance as the monoepoxide TAG51:1 (O) was present in higher concentrations than TAG54:2 (O) and TAG54:3 (O) in both margarines, M2 and M3.

Summarized, the oxidative stability of margarines was evaluated using the peroxide value, conjugated dienes, the Rancimat method and oxTAGS. Whereas the peroxide value and the conjugated dienes detect primary lipid oxidation products, the Rancimat method was used to detect secondary lipid oxidation products. The results of the different methods matched well together, showing decreasing oxidative stability of margarine during storage. Hence, the analyzed results were taken as the basis for the screening of new possible indicators to detect the oxidation status of margarine.

### 3.3. Screening of Volatile Products in Stored Margarine

**Figure 2:**
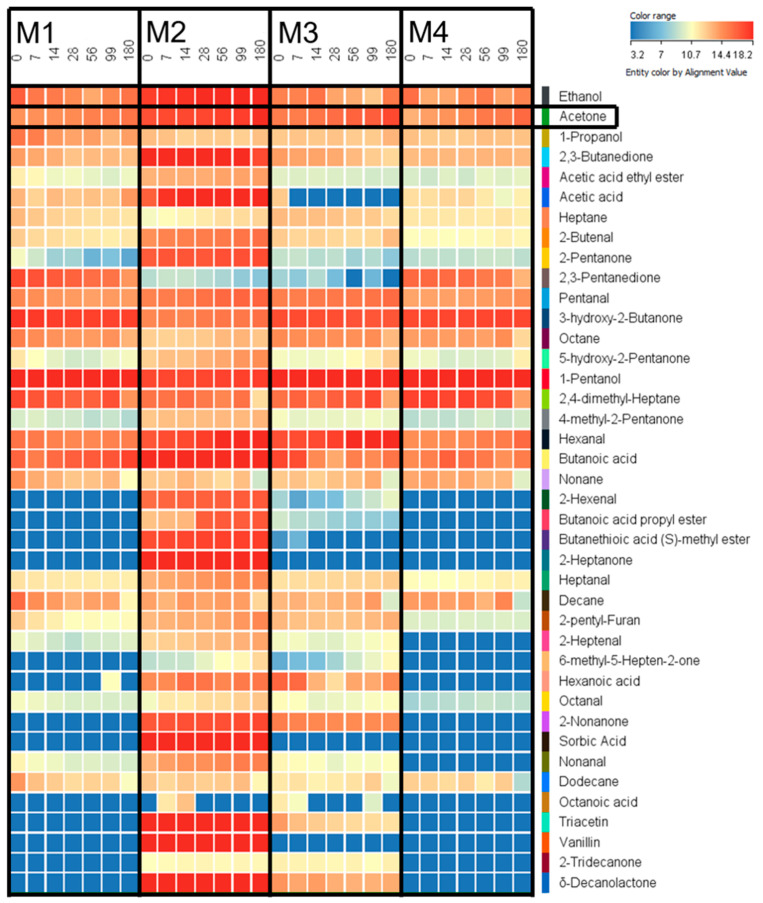
Heatmap comparing the relative intensity of volatile compounds in the different margarines M1–M4 between day zero and day 180 of storage. Low intensities are marked in blue color, middle intensities in yellow color and high intensities in red color.

The four different margarines were analyzed via HS-SPME-GC-MS to screen for volatile products that form during storage. Differences between the margarine types were evaluated and are depicted as heatmap (Figure 2). The heatmap shows that the content of volatile compounds was much higher in M2 than in the other margarine types. It also shows that the samples M1 and M4 had a similar spectrum of volatile compounds, which could be confirmed by the similar fatty acid composition of these products (Table 1, Figure 1).

Margarine M2 was the only margarine that contained 2-pentanone, 5-hydroxy-2-pentanone, 4 methyl-2-pentanone, 2-hexenal, butanoic acid propyl ester, butanethioic acid methyl ester and 2 heptanone. However, M2 was the only margarine that contained anhydrous milk fat and yogurt culture from skimmed milk. 2-Pentanone and 2-heptanone are methyl ketones that derive from short-chain and middle-chain fatty acid that exhibit less than 14 C-atoms and are typically found in milk fats. They form through enzymatic hydrolyzation and a sideway of the β-oxidation of triacylglycerides. Autoxidation of α-linolenic acid generates the aldehydes 2,4-heptadienal and 2,4,7 decatrienal which can lead to the formation of 2-hexenal through further autoxidation [37]. Butanoic acid propyl ester and butanethioic acid (S)-methyl ester are typically found in milk fats [29] and were therefore only found in M2, which contained anhydrous milk fat. Butanoic acid, which was found in all four different margarines, might derive from the butter aroma, which was added to each margarine (Table 1) [38].

In M1 and M4, 2,3-pentanedione was detected exclusively and could neither be found in M2 nor M3. This diketone might be derived from sunflower oil [39], which was only used as an ingredient in M1 and M4.

The heatmap led to the assumption that acetone and hexanal were the only volatiles that significantly increased in all four types of margarine during storage. As acetone has not been described in margarine so far, a quantitative HS-SPME-GC-MS analysis of the herein studied margarine was performed to analyze its impact during lipid oxidation.

### 3.4. Acetone Increased during Lipid Oxidation in Margarine

**Figure 3:**
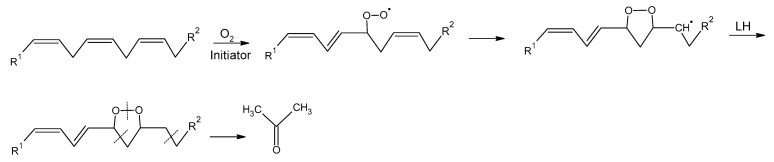
Proposed mechanism of the formation of acetone during lipid oxidation.

As the analysis of the heatmap indicated that acetone increased during a storage period of 180 days in margarine, the question arose how acetone is formed during lipid oxidation and whether it could be used as a potential new indicator for lipid oxidation. Figure 3 describes a proposed mechanism of the formation of acetone during lipid oxidation. Unsaturated fatty acids might be oxidized to peroxyl radicals followed by a cyclization leading to the formation of epidioxide radicals. The addition of the peroxyl radical to the first carbon of the β-double bond is known to compete with H abstraction depending on temperature and oxygen pressure [40]. The epidioxide radicals might get reduced to epidioxides which might decompose to acetone through fragmentation by C-C and O-O bond cleavage.

**Figure 4:**
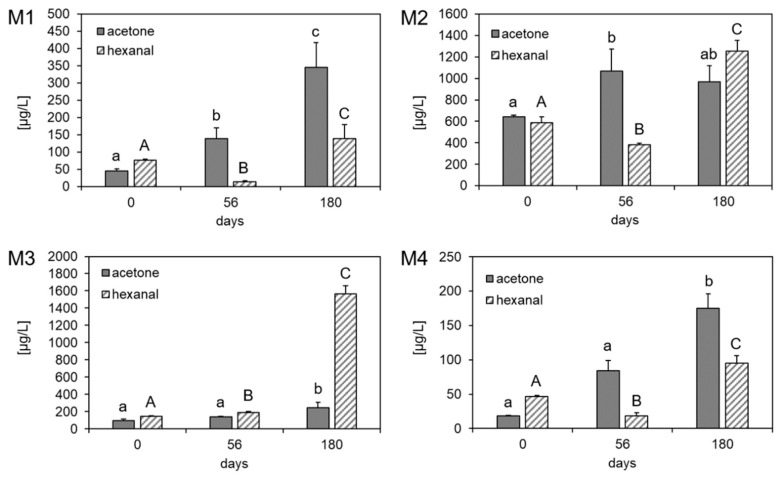
Concentrations of acetone (µg/L) and hexanal (µg/L) in the different margarine types M1–M4 over the storage period of 180 days. Data are depicted as mean + SD (n = 3). Statistically significant differences between the concentration of acetone during storage are indicated with different lowercase letters (a, b, c). Statistically significant differences between the concentration of hexanal during storage are indicated with different capital letters (A, B, C) (one-way ANOVA, Holm–Sidak, *p* < 0.05).

A standard addition experiment was performed to calculate the concentrations of acetone and hexanal at days 0, 56, and 180. Hexanal, a decomposition product of linoleic acid [37], is a recognized marker for lipid oxidation and was therefore monitored as well. The results are shown in Figure 4.

It could be demonstrated that acetone and hexanal increased in all types of margarine within 180 days. Hexanal showed fluctuating values, that, except of M3, decreased after 56 days and increased from 56 days to 180 days. This fluctuation of hexanal during storage has been shown elsewhere [41,42], as the rate of peroxide formation might not be equal to the rate of peroxide decomposition, thereby leading to the fluctuating hexanal values [42].

Acetone increased from 45.4 ± 5.80 µg/L to 345 ± 71.5 µg/L in M1, from 641 ± 17.3 µg/L to 968 ± 149 µg/L in M2, from 98.2 ± 13.7 µg/L to 243 ± 62.0 µg/L in M3 and from 18.3 ± 0.60 µg/L to 175 ± 21.0 µg/L in M4. M2 exhibited the highest concentration of acetone after 180 days, followed by M1 and M3. M4 showed lower concentrations of acetone compared to the other margarines. The concentrations of acetone in M1 and M4 at days 56 and 180 were even higher than the concentrations of hexanal, thereby confirming its possible application as an indicator for lipid oxidation in margarine.

Regarding the variation in acetone changes, which differ more than tenfold between the different margarines, it has to be noted that the extent of acetone formation might strongly depend on the different margarine matrices. We further hypothesize that the formation of acetone proceeds through a different pathway than the formation of hexanal and that both pathways are in competition. Whereas acetone might be formed through epidioxides, hexanal is formed through H abstraction pathway [43].

It is well known that a too narrowly-defined profile of lipid oxidation products for monitoring deterioration of lipids, especially by using only a single marker, might lead to an underestimation of lipid oxidation or even miss it when alternate reactions occur. The analytical challenge is to combine chemical analyses that monitor all different pathways [35]. The advantage of quantitating acetone as a new possible marker is that a new pathway, which has not been regarded so far, will be taken into consideration, although this method requires more expensive equipment than measuring the peroxide value or conjugated dienes Further, the formation of acetone in margarine reveals the need for careful choice of oxidation parameters in antioxidant studies when applied to *W*/*O* systems.

By comparing the acetone concentrations with the commonly applied methods, such as peroxide value, UV absorption of conjugated dienes and Rancimat method, it could be shown that the results confirm the increase and change of lipid oxidation markers during storage. The acetone content of M1 and M4 changed during storage by about 660 and 856%, respectively, whereas in M2 and M3 a change of the acetone concentration by about 51.0 and 147% could be demonstrated. The same picture emerged for the results of the peroxides and conjugated dienes, where in M1 and M4 higher changes during storage were exhibited than in M2 and M3. Further, the OIT value fitted to these results as M2 and M3 were shown to be more resistant against oxidation than M1 and M4. To confirm the results, a correlation analysis between the acetone concentration in the stored margarine samples and the corresponding content of unsaturated fatty acids, peroxide value, conjugated dienes and the OIT was performed. Acetone correlated with the unsaturated fatty acids (correlation coefficient: 0.777, *p* < 0.01), the peroxide value (correlation coefficient: 0.784, *p* < 0.001) and the conjugated dienes (correlation coefficient: 0.878, *p* < 0.001). Acetone did not correlate with the OIT (correlation coefficient: −0.193, *p* = 0.265), even though higher acetone values tended to cause lower OIT values. This might indicate a better correlation of acetone with primary lipid oxidation markers thereby corroborating the proposed pathway of the formation of acetone through hydrogen abstraction-independent reactions.

To limit the extent of acetone, the use of antioxidants would be beneficial. Antioxidants have the potential to decelerate or stop the oxidation of other substances by acting as radical scavengers, singlet oxygen quenchers or through UV absorption [44,45,46,47]. In emulsions, such as margarine, the polarity [48,49] and the chain length [50] of the antioxidants are of great importance. While hydrophilic, polar antioxidants, such as green tea extract, exhibit stronger impact on the oil phase and are more effective in W/O emulsions, lipophilic, nonpolar antioxidants, such as tocopherols, have higher impact on aqueous phases and are more active in *W*/*O* emulsions [48,49], a phenomenon that is known as “polar paradox” [49]. Laguerre et al. [50] showed that also the cutoff effect [51] plays a crucial role in the antioxidative efficiency of antioxidants. They investigated the relationship between antioxidant property and hydrophobicity by using a complete homologous series of chlorogenic acid esters. Thereby, the authors observed that the antioxidant capacity increased with increasing alkyl chain length until reaching the length of the dodecyl chain and that further extension of the chain led to a drastic decrease in the anti-oxidative efficiency [50].

To the best of our knowledge, this is the first study showing the formation of acetone in margarine during storage. Literature shows scarce results regarding the formation of acetone during lipid oxidation. Xia and Budge [9] described that ketones form during lipid oxidation from an unsaturated site in triacylglycerols, whereas Timmins et al. [52] postulated that triplet acetone results from the cyclization of the alkyl peroxyl radical to a dioxetane radical intermediate followed by its thermolysis. Further, Amlendu et al. [10] described breath acetone as a biomarker for lipid oxidation in humans, while Mano et al. [53] detected 33 distinct carbonyl-containing compounds by using a HPLC equipped with a photodiode array detector and a Fourier transform ion cyclotron mass spectrometer. They compared these 33 compounds between two lines of Arabidopsis, which displayed different fatty acid compositions—the wild type Col-0 and the fad7fad8 double mutant, which lacked trienoic fatty acids in the plastid. After the comparison, they showed that main differences could be detected in the concentrations of malondialdehyd, acrolein, (E)-2-pentenal, 3 pentenanone, n-hexanal, and acetone [54], whereby the fad7fad8 mutant showed higher amounts of 3-pentanone, n-hexanal, and acetone [53]. This difference in the carbonyl composition agreed with the oxidative degradation of dienoic and trienoic fatty acids in vitro [53]. Interestingly, they revealed that the two types of *A. thaliana* showed differences in their very short chain carbonyls, such as acetone, formaldehyde and acetaldehyde. The leaves of the fad7fad8 mutant type contained 29% greater amounts of acetone, 30% less formaldehyde and 16% less acetaldehyde. The production of these carbonyls in plants has been described due to the alcohol and organic acid metabolism [55], but the results from Mano et al. [53] suggested that the oxidation of fatty acids significantly contributed to their formation.

However, the formation of acetone in margarines or oils during lipid oxidation has not been described so far. The here presented results suggest that acetone could be used as an indicator for lipid oxidation in margarine. Further analyses are warranted to confirm the use of acetone as a new possible marker for lipid oxidation in margarine and other food lipids.

## 4. Conclusions

In this study, four different margarines were stored at 15 °C for 180 days and analyzed regarding peroxide value, conjugated dienes, oxidative stability and oxTAGs at days 0, 1, 7, 14, 28, 56, 99, and 180. Further, the different margarines were analyzed by SPME-GC-MS to detect volatiles which formed during storage. Results showed that the fatty acid profile of each margarine influenced its oxidative stability and the formation of different volatiles. The content of volatile compounds was the highest in M2, whereas M1 and M4 had a similar profile of volatile compounds, which could be confirmed by the similar fatty acid composition of these margarine samples. The HS-SPME-GC-MS analysis revealed that acetone and hexanal increased in all types of margarines during storage. Acetone, which was proposed to form through cyclization of peroxyl radicals followed by a reduction step and C-C and O-O bond cleavage, could be detected in margarine for the first time. The concentration of acetone correlated well to the results of the unsaturated fatty acids, peroxide value and conjugated dienes, thereby demonstrating the possible use of acetone as a new marker for lipid oxidation in margarine.

## Figures and Tables

**Table 1:** Composition of margarine samples M1, M2, M3 and M4.

	M1	M2	M3	M4
Oils	32% hydrogenated coconut oil	51% palm oil	57% palm oil	32% hydrogenated coconut oil
	22% coconut oil	10% palm oil—free of trans fatty acids, non-hydrogenated	12% palm stearin	24% coconut oil
	13% sunflower oil	8% anhydrous milk fat	11% rapeseed oil	14% sunflower oil
	10% palm oil or transesterified palm kernel oil	7% rapeseed oil		6% interesterified blend of coconut oil and fully hydrogenated rapeseed oil
	3% rapeseed oil	4% coconut oil		3% rapeseed oil
Water	20%	19%	20%	20%
Minor ingredients	0.30% Palsgaar P1388 (mono- and diglycerides of edible fatty acids and polyglycerol esters)	1% yogurt culture from skimmed milk	0.18% Dimodan HP (distilled monoglyceride)	0.30% Palsgaar P1388 (mono- and diglycerides of edible fatty acids and polyglycerol esters)
	0.10% salt non-iodized	0.35% Dimodan HP (distilled monoglyceride)	0.10% salt non-iodized	0.10% salt non-iodized
	0.01% butter aroma	0.11% butter aroma	0.08% lecithin	0.01% butter aroma
	0.01% citric acid	0.10% salt non-iodized	0.04% butter aroma	0.01% citric acid
	0.001% β-carotene	0.08% lecithin	0.01% citric acid	0.001% β-carotene
		0.06% citric acid	0.001% β-carotene	
		0.04% potassium sorbate		
		0.001% β-carotene		

**Table 2:** Oxidative stability of margarine during a storage period of 180 days at 15 °C analyzed by peroxide value (meq O_2_/kg oil), conjugated dienes and the Rancimat method (h). Data are depicted as mean ± SD (n = 4). The asterisks show a significant difference to day 0 (one-way ANOVA, Holm-Sidak, *p* < 0.05).

**−**	**Day**	**M1 (meq O_2_/kg Oil)**	**Change (%)**	**M2 (meq O_2_/kg Oil)**	**Change (%)**	**M3 (meq O_2_/kg Oil)**	**Change (%)**	**M4 (meq O_2_/kg Oil)**	**Change (%)**
peroxide value	0	0.33 ± 0.02		1.10 ± 0.10		0.86 ± 0.06		0.40 ± 0.03	
	1	0.36 ± 0.05	9.09	1.23 ± 0.10	11.8	0.89 ± 0.09	3.49	0.40 ± 0.04	0.00
	7	0.41 ± 0.06	24.2	1.31 ± 0.14	19.1	0.94 ± 0.10	9.30	0.46 ± 0.0.5	15.0
	14	0.43 ± 0.01	30.3	1.36 ± 0.23	23.6	0.97 ± 0.23	12.8	0.44 ± 0.03	10.0
	28	0.56 ± 0.08	69.7	2.37 ± 0.22	115	1.24 ± 0.05	44.2	0.60 ± 0.05	50.0
	56	0.82 ± 0.07	148	3.42 ± 0.28 *	211	1.93 ± 0.38	124	0.99 ± 0.06	148
	99	1.31 ± 0.21 *	297	4.08 ± 0.26 *	271	3.06 ± 0.34 *	256	1.64 ± 0.28 *	310
	180	2.20 ± 0.67 *	567	4.76 ± 0.92 *	333	3.97 ± 0.51 *	362	2.73 ± 0.27 *	583
	**Day**	**M1 (µmol/g)**	**Change (%)**	**M2 (µmol/g)**	**Change (%)**	**M3 (µmol/g)**	**Change (%)**	**M4 (µmol/g)**	**Change (%)**
conjugated dienes	0	4.45 ± 0.21		11.2 ± 0.68		7.75 ± 0.42		5.42 ± 0.14	
	1	4.66 ± 0.15	4.72	11.3 ± 0.25	0.89	7.69 ± 0.36	−0.77	3.75 ± 0.61 *	−30.8
	7	4.98 ± 0.40	11.9	11.3 ± 0.08	0.89	7.59 ± 0.19	−2.06	5.47 ± 0.27	0.92
	14	5.70 ± 0.25 *	28.1	10.7 ± 0.44	−4.46	8.14 ± 0.56	5.03	5.33 ± 0.62	−1.66
	28	5.23 ± 0.71	17.5	12.2 ± 0.20	8.93	7.81 ± 0.48	0.77	5.29 ± 0.43	−2.40
	56	5.80 ± 0.60 *	30.3	12.6 ± 0.23	12.5	8.50 ± 0.47	9.68	6.31 ± 0.12	16.4
	99	6.12 ± 0.90 *	37.5	12.3 ± 0.42	9.82	9.96 ± 0.20 *	28.5	5.60 ± 0.59	3.32
	180	8.35 ± 0.59 *	87.6	14.7 ± 0.49 *	31.3	9.98 ± 0.73 *	28.8	7.89 ± 0.71 *	45.6
	**Day**	**M1 (h)**	**Change (%)**	**M2 (h)**	**Change (%)**	**M3 (h)**	**Change (%)**	**M4 (h)**	**Change (%)**
oxidation induction time	0	10.3 ± 0.22		13.0 ± 0.23		11.7 ± 0.37		10.7 ± 0.09	
	1	11.0 ± 0.88	6.80	13.2 ± 0.07	1.54	11.5 ± 0.11	−1.71	10.2 ± 0.07	−4.67
	7	10.1 ± 0.23	−1.94	12.6 ± 0.15	−3.08	11.4 ± 0.15	−2.56	9.61 ± 0.09	−10.2
	14	9.39 ± 0.02	−8.83	12.2 ± 0.39	−6.15	10.9 ± 0.20 *	−6.84	8.99 ± 0.02	−16.0
	28	9.64 ± 1.09	−6.41	11.1 ± 0.16	−14.6	10.5 ± 0.11 *	−10.3	8.23 ± 0.37	−23.1
	56	8.02 ± 0.02	−22.1	9.98 ± 0.19	−23.2	9.87 ± 0.10 *	−15.6	7.44 ± 0.04	−30.5
	99	6.83 ± 0.11	−33.7	9.79 ± 0.44 *	−24.7	8.93 ± 0.17 *	−23.7	6.50 ± 0.22 *	−39.3
	180	5.55 ± 0.01 *	−46.1	7.96 ± 0.15 *	−38.8	7.71 ± 0.20 *	−34.1	5.25 ± 0.20 *	−50.9

**Table 3:** Epoxidized TAGs 54:1 (O), 54:2 (O) and 54:3 (O) (mg epoxide/kg margarine) in margarines M2 and M3.

**M2**	**Day 0 (mg Epoxide/kg Margarine)**	**Day 180 (mg Epoxide/kg Margarine)**
54:1 [O]	0.58 ± 0.03	0.24 ± 0.06
54:2 [O]	0.19 ± 0.01	0.08 ± 0.06
**M3**	**Day 0 (mg Epoxide/kg Margarine)**	**Day 180 (mg Epoxide/kg Margarine)**
54:1 [O]	1.06 ± 0.10	1.59 ± 0.33
54:2 [O]	0.77 ± 0.11	1.32 ± 0.33
54:3 [O]	0.07 ± 0.04	0.27 ± 0.16

## Data Availability

Data is contained within the article.
